# Circadian rhythms and breast cancer: unraveling the biological clock’s role in tumor microenvironment and ageing

**DOI:** 10.3389/fimmu.2024.1444426

**Published:** 2024-07-30

**Authors:** Yalan Yan, Lanqian Su, Shanshan Huang, Qihui He, Jiaan Lu, Huiyan Luo, Ke Xu, Guanhu Yang, Shangke Huang, Hao Chi

**Affiliations:** ^1^ Clinical Medical College, Southwest Medical University, Luzhou, China; ^2^ Department of Paediatrics, Southwest Medical University, Luzhou, China; ^3^ Department of Oncology, Chongqing General Hospital, Chongqing University, Chongqing, China; ^4^ Department of Specialty Medicine, Ohio University, Athens, OH, United States; ^5^ Department of Oncology, The Affiliated Hospital, Southwest Medical University, Luzhou, China

**Keywords:** circadian rhythm, breast cancer, therapeutic targets in immunotherapy, aging, tumor microenvironment, clockwork therapy, tumor-immune interactions

## Abstract

Breast cancer (BC) is one of the most common and fatal malignancies among women worldwide. Circadian rhythms have emerged in recent studies as being involved in the pathogenesis of breast cancer. In this paper, we reviewed the molecular mechanisms by which the dysregulation of the circadian genes impacts the development of BC, focusing on the critical clock genes, brain and muscle ARNT-like protein 1 (BMAL1) and circadian locomotor output cycles kaput (CLOCK). We discussed how the circadian rhythm disruption (CRD) changes the tumor microenvironment (TME), immune responses, inflammation, and angiogenesis. The CRD compromises immune surveillance and features and activities of immune effectors, including CD8+ T cells and tumor-associated macrophages, that are important in an effective anti-tumor response. Meanwhile, in this review, we discuss bidirectional interactions: age and circadian rhythms, aging further increases the risk of breast cancer through reduced vasoactive intestinal polypeptide (VIP), affecting suprachiasmatic nucleus (SCN) synchronization, reduced ability to repair damaged DNA, and weakened immunity. These complex interplays open new avenues toward targeted therapies by the combination of clock drugs with chronotherapy to potentiate the immune response while reducing tumor progression for better breast cancer outcomes. This review tries to cover the broad area of emerging knowledge on the tumor-immune nexus affected by the circadian rhythm in breast cancer.

## 1 Introduction

From gene expression and cellular metabolism to intricate biological behaviors, endogenous oscillations in organisms over approximately 24 hours are known as circadian rhythms. Disruptions in these rhythms increase the risk of various cancers (1, 2). In the breast, altered circadian gene expression affects breast biology, potentially promoting cancer (3). The coordination of intrinsic molecular clock networks between central and peripheral tissues maintains circadian rhythms. Core genes such as BMAL1 and CLOCK are positive transcription factors in circadian rhythms, which are involved in the regulation of immune cell function (4), and their overexpression promotes cancer cell proliferation and invasion (5, 6). Circadian rhythms also adjust the TME with tumor initiation and influence (7), and the tumor microenvironment is closely related to multiple stages of tumor initiation, progression, invasion, metastatic spread and growth (8). In this, proliferative and invasive behaviors are crucial processes.

Peripheral tissue rhythms are coordinated by the central clock in the SCN (9). Aging impairs SCN function, promoting age-related diseases (10). Breast cancer, an age-related disease, illustrates the complex interplay between circadian disruption, aging, and cancer risk. Circadian rhythm-related studies propose strategies for combining clock drugs and therapies to treat breast cancer.

This review outlines the role of circadian genes in breast cancer development, the impact of CRD on the TME, and the relationship between circadian rhythms, aging, and cancer risk. By synthesizing these findings, we aim to reveal the significant role of circadian rhythms in breast cancer pathogenesis and provide new treatment perspectives.

## 2 Exploring the relationship between circadian rhythms and breast cancer

### 2.1 Basis of circadian rhythms

**Figure 1:**
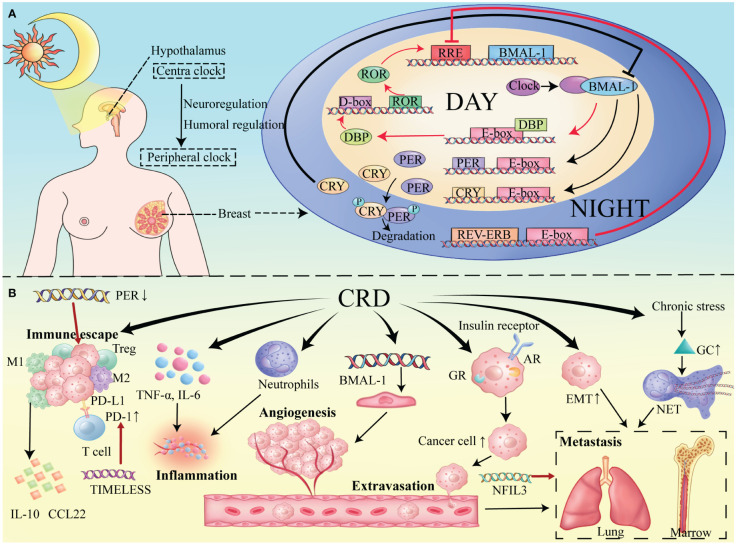
**(A)** Circadian Molecular Mechanism in Mammary Tissue. Main Loop (black lines): CLOCK-BMAL1 induces the expression of PER and CRY proteins. The main pathway for the production and maintenance of circadian rhythm. Secondary Loop (red lines): ROR and REV-ERB respectively promote and inhibit the production of BMAL1, thereby maintaining the stability of the main loop. **(B)** Impact of the tumor microenvironment on breast cancer under conditions of circadian rhythm disruption.

The mammalian circadian system is multileveled and includes a central clock located in the hypothalamic SCN along with distributed peripheral clocks in the various tissues (11). (**Figure 1A**) The SCN nuclei maintain circadian rhythms, with a significant input pathway from environmental light signals (12). Light information is conveyed from the retina to the SCN via the retinal-hypothalamic pathway (13). Although peripheral clocks can autonomously generate rhythms, synchronization with the central clock is essential for coherence (14). This synchronization occurs through neural and humoral pathways, maintaining consistent phase relationships within the circadian system (11).

At the molecular level, a complex network of transcription factors forms at the heart of the mammalian circadian clock, interacting through various transcriptional and translational feedback loops (TTFLs) that autoregulate activation and inhibition, thereby imposing the 24-hour cyclic rhythm on this process (15) (**Figure 1A**).

Within the primary loop of the circadian cycle, the positive regulators are heterodimers composed of BMAL1 and CLOCK transcription factors. During daylight hours, the CLOCK-BMAL1 complex binds to E-box regions in target genes, promoting their expression. These genes include negative regulators such as Period (PER) and Cryptochrome (CRY), which modulate the circadian rhythm. At night, PER and CRY form heterodimers, translocate to the nucleus, and inhibit CLOCK-BMAL1 activity, halting transcription (16, 17). Post-translational modifications then lead to PER and CRY degradation (18), allowing the cycle to restart once their levels are sufficiently reduced.

In addition to PER and CRY, the CLOCK-BMAL1 complex targets orphan nuclear receptors REV-ERBα and REV-ERBβ (19). These receptors are integral to forming a secondary feedback loop with RORα, RORβ, and RORγ (20). REV-ERB and ROR proteins compete for binding to the RRE elements in target gene promoters and enhancers, modulating transcription and ensuring the rhythmic expression of BMAL1 (21).

Other TTFLs include key transcription factors like DBP, which contribute to the stability of the circadian clock, maintaining oscillations even in constant conditions (22).

### 2.2 Circadian rhythm genes involved in breast cancer development

Circadian rhythms are regulated by a series of clock genes, which are strongly associated with the progression of breast cancer.

BMAL1 and CLOCK, as key transcription factors, are integral to breast cancer progression. Their overexpression correlates with increased tumor cell proliferation and invasion (5, 6). Notably BMAL1 regulates the involvement of Nrf2 in carcinogenesis, for example, its deletion downregulates the Nrf2-mediated antioxidant pathway and significantly increases the amount of IL-1β, which promotes cancer (5). Whereas, Nrf2 has both oncogenic and carcinogenic effects in breast cancer, and its oncogenic effects are achieved by binding to keap-1 to reduce Reactive Oxygen Species (ROS) and inhibit the pro-cancer effects of high levels of ROS (23). Furthermore, c-Myc can regulate cell proliferation (24), and is a key mediator between breast cancer cells and TME (25). And the target E-box sequence of CLOCK-NPAS2 (neuronal PAS domain protein 2)-BMAL1 complex is also a c-Myc cross site. Therefore, the core clock gene may regulate c-Myc expression to regulate cell proliferation (26).

Conversely, the PER and CRY genes act as negative regulator of circadian rhythms. Deletion of PER2 enhances breast cancer cell proliferation, likely due to decreased p53, increased c-Myc and increased CyclinD1 expression (18). PER2 is considered to be a tumor suppressor by inhibiting Epithelial-Mesenchymal Transition (EMT) and controlling cell proliferation; however, the ZnF704/SIN3A complex inhibits PER2 transcription, disrupts circadian rhythms, and exacerbates cancer cell invasion and metastasis (27). Recent studies have shown that CRY1 inhibits the growth of triple-negative breast cancer (TNBC) cells, which is associated with its inhibition of Pyruvate Dehydrogenase Kinase 1 (Pdk1) expression, Pyruvate Dehydrogenase (PDH) phosphorylation, and glucose depletion (28). In addition to this, CRY1 is involved in cellular DNA damage repair, and its depletion enhances DNA damage in cancer cells, whereas CRY1 expression is reduced in the presence of Yes-associated protein (YAP) silencing or TEA domain transcription factor (TEAD) inhibition (29). As for CRY2, it can exert an anti-proliferative effect on breast cancer cells because it may bind and inhibit the p65/p50 complex, thus inhibiting the nuclear factor-κB (NF-κB) pathway, but its acetylation in breast cancer impairs this function (30), the exact mechanism has yet to be studied. These findings underscore the complex relationship between circadian genes and breast cancer development.

Moreover, other circadian components are crucial. For instance, elevated expression of nuclear factor, interleukin 3 regulated (NFIL3) enhances TNBC cell proliferation and metastasis by inhibiting Nuclear Factor kappa B Inhibitor Alpha (NFκBIA) transcription and boosting NF-κB signaling (31). Similarly, TIM (TIMLESS) facilitates immune evasion by upregulating PD-1, which suppresses CD8+ T cell immunoreactivity (32). And hypomethylation of the TIM promoter correlates with advanced breast cancer (33). Overall, TIM is significantly associated with clinical prognosis across various cancers (34, 35) (**Table 1**).

**Table 1:** Circadian rhythm genes in breast cancer.

Character	Genes	Mechanism	Effect on breast cancer	reference
Positive	CLOCK-NPAS2-BMAL1	Control the expression of oncogene c-Myc	Regulates cell proliferation in breast cancer	(26)
	BMAL1	Direct regulation of Nrf2	Regulation of the Dual Role of Nrf2 in Breast Cancer	(5)
Negative	PER2	When deficient, p53 expression decreases, while the expression of c-Myc and its target cell Cyclin D1 increases	Promote proliferation of breast cancer cells	(18)
	CRY1	Inhibition of Pdk1 expression, PDH phosphorylation, and glucose consumption	Inhibition of TNBC cell growth	(28)
	CRY1	Overactivation of YAP and enhanced DNA damage response	Altered cell growth in breast cancer	(29)
	CRY2	Acetylation	The antiproliferative effect is diminished in breast cancer	(30)
Other	NFIL3	Inhibit the transcription of NEκBIA and enhance NF-κB signaling.	Promote TNBC cell proliferation and metastasis	(31)
	TIMELESS	Up-regulated the expression of PD-L1 and inhibited the immune activity of CD8T cells	Promotes immune evasion in breast cancer	(32)

### 2.3 TME in the context of CRD

CD8+ T cells, M1 macrophages, and neutrophils perform immune surveillance in the process of irruption through the stages of breast cancer. Meanwhile, suppressive cells, such as M2 macrophages, myeloid-derived suppressor cells, and regulatory T cells, have indispensable roles in the tumor microenvironment, creating a complex network of immune responses in the tumor (36). Cancer cells often have increased stemness, while T cells predominantly exhibit a regulatory or exhausted phenotype, and the macrophages a type M2 phenotype (37, 38).

Circadian clocks regulate the tumor microenvironment of breast cancer through complex mechanisms of immune cell type and activity, inflammation, angiogenesis, and tumor cell migration and invasion, all constituting central features of the breast cancer microenvironment (**Figure 1B**).

M2-type tumor-associated macrophages (TAMs) promote immune tolerance by producing anti-inflammatory cytokines, such as CCL22 and IL10 (39). Under conditions of CRD, both M1-type macrophages and M1/M2 ratios were reduced in the tumor, the proportion of regulatory T cells was increased, and myeloid cell infiltration (7, 40, 41). Shift in cytokine production balance from Th1-cytokines to Th2 cytokines (including IL-10) (42). And dysregulated clock gene expression was positively correlated with the levels of T-cell incompetence markers such as programmed cell death protein 1, all of which promoted immune escape (15).

Inflammation is a crucial driver of tumor progression, promoting the upregulation of pro-inflammatory cytokines under chronic jet lag, such as TNF-α and IL-6, which modulate the disruption of circadian rhythms (43). CRD can worsen neutrophil-driven inflammation, further degrading the tumor microenvironment (44). Circadian rhythm genes also regulate angiogenesis. Endothelial cell formation, the starting point for angiogenesis, is affected by circadian genes like BMAL1 (45) which regulate the endothelial cell cycle and thus impact angiogenesis and tumor progression (46). In a spontaneous mouse model of breast cancer, circadian rhythm disruption promotes cancer cell spread and metastasis (47). This may be due to elevated expression of EMT-related genes under CRD conditions, making cancer cells more motile and invasive (48, 49). CRD-associated chronic stress releases glucocorticoids, forming neutrophil extracellular traps (NETs) that foster a metastasis-promoting environment (50, 51). Breast cancer frequently metastasizes to the bone and lungs (52, 53). Circulating tumor cell (CTC) exudation predominantly occurs during sleep (54), influenced by circadian-regulated hormone receptors, which increases CTC activity and impacts breast cancer progression (55, 56). An in-depth study of the finer and more specific mechanisms by which CRD regulates TME can help provide a clearer direction for research.

## 3 Exploring aging, circadian rhythms, and breast cancer risk

Circadian rhythms are crucial for regulating immune cell activity, immune responses, and inflammation (15). Breast cancer is an age-related disease, which significantly contributes to its risk factors. Evidence demonstrates that this relationship is bidirectional (57); in other words, aging through disruption of the circadian rhythm is accelerated, and vice versa (58). These interactions are closely linked to breast cancer progression.

Aging disrupts circadian rhythms in several ways. First, it weakens light signal entrainment and diminishes neuronal electrical activity and communication. Light transmission through the lens and pupil can decrease by up to 90% (59), leading to desynchronization within the central clock and peripheral clocks (60). Additionally, SCN neuronal activity declines with age, reducing the rhythmic amplitude and coherence of firing patterns, causing phase desynchronization (61). Disruption of the biological clock in aging mammals has been linked to significantly impaired interneuronal communication in the SCN. Although the total number of neurons within the SCN remains relatively constant during aging, there is a marked decrease in the subpopulation that secretes the neurotransmitter VIP (62). VIP is an important mediator of interneuronal coupling in the SCN, and their decrease leads to a reduction in cell-to-cell coupling and thus affects the synchronization of the SCN network (60). On the other hand, aging also affects the peripheral clock, but the exact mechanism of action remains unclear, one reason being that different tissues and organs change differently during aging (63).

Similarly, the circadian system influences aging. In drosophila and mice, system-wide knockouts of CLOCK genes (including BMAL1, clock, PER, etc.) produce an “accelerated aging” phenotype, suggesting that central and peripheral molecular clocks play an important role in aging (64). Alterations independent of the loss of circadian rhythms in behavior. It has also been proposed that Bmal1 also interacts with the nuclear factor-activated κ-light chain-enhanced (NF-κB) signaling system in B cells, and that knockdown of this gene leads to a chronic inflammatory state, which accelerates the senescence phenotype (58).

Aging is a significant risk factor for breast cancer development (65). The combination of increased endogenous and exogenous DNA damage (66) and reduced DNA repair capacity in aging cells raises the likelihood of mutations in critical mammary cell genes (67). For instance, mutations in BRCA1 and BRCA2 during aging can increase tumor development risk by 80% (68). Aging also weakens the immune system, reducing its ability to detect and eliminate cancerous cells, leading to tumorigenesis (69). This decline in immune function causes chronic inflammation (70), associated with various cancers, including breast cancer (71) and prostate cancer (72). Additionally, aging affects estrogen levels, crucial for breast growth and cancer progression (73). As individuals age, the proportion of adipocytes producing estrogen rises, leading to sustained high levels of estradiol. This, in turn, affects the expression of estrogen receptors and the behavior of cancer cells (74).

## 4 Conclusion and prospect

From the preceding discussion, it is evident that disruptions in circadian rhythms are strongly linked to the initiation and advancement of breast cancer. Consequently, we are proposing a pioneering therapeutic approach for breast cancer that leverages circadian rhythms. It is well-recognized that conventional breast cancer treatments primarily impede disease progression through various strategies: adjusting estrogen receptor levels, disrupting DNA and RNA synthesis in cancer cells, inhibiting cyclins, and specifically targeting the HER2 receptor. This novel method focuses on circadian rhythms, aiming to achieve therapeutic outcomes by correcting disrupted circadian rhythms at the cellular or molecular level. Moreover, this treatment offers significant advantages over traditional methods, including enhanced drug effectiveness, fewer side effects, and improved immune response (75). This emerging focus has garnered significant interest. There are two significant strategies being followed at present for the treatment of breast cancer based on circadian rhythm: 1) the administration of drugs either directly or indirectly acting on components of biological clocks, and 2) scheduling drug administration according to the circadian host’s endogenous clock.

Clock drugs exert their anticancer effects both by the direct modulation of the circadian rhythm, such as through the biological clock by core circadian genes, and indirectly by the rhythm gene regulators. The latter method focuses on proteins that are involved in the phosphorylation or degradation of clock components, such as REV-ERB, ROAR, CRY1/2, CRA, and the casein kinase (CK) family (18), which is more straightforward and potent. For instance, CRY protein modulators inhibit breast cancer cell proliferation without affecting normal breast epithelial cells (76). These pharmaceutical interventions have brought new hope for breast cancer management (77). However, challenges remain due to limited human research on circadian gene expression in breast cancer (78) and the low specificity of clock drugs (1), affecting their clinical application.

Chronotherapy, which involves administering treatment at specific times aligned with circadian rhythms, enhances efficacy and reduces side effects, especially in cancer therapy by lowering toxicity and increasing drug effectiveness (79, 80). Experiments in rodents have shown that the timing of anticancer drugs can result in 2- to 10-fold differences in tolerance (81). Clinical trials, such as those using azithromycin and cisplatin in advanced ovarian cancer, have demonstrated increased survival rates (82). However, a study in rectal cancer found no significant benefit from clock-based drug administration (83). Thus, the evidence for routine clinical use of chronotherapy in cancer treatment remains inconclusive. Additionally, breast cancer chronotherapy faces challenges like the unclear oscillation mechanisms in breast tissues and the absence of effective methods to detect breast clock gene expression patterns (84). Future strategies must carefully consider drug timing design (26).

In addition, regarding the diagnosis and prognosis assessment of breast cancer, conducting research from the perspective of circadian rhythms, developing biomarkers related to circadian rhythms, and establishing a prognosis assessment system based on circadian rhythms … are all worthwhile research areas to explore.

## Author contributions

YY: Writing – original draft, Writing – review & editing, Data curation. LS: Data curation, Writing – original draft, Writing – review & editing. SSH: Writing – original draft. QH: Writing – original draft. JL: Writing – original draft. HL: Writing – original draft. KX: Writing – original draft. GY: Supervision, Writing – original draft. SH: Funding acquisition, Supervision, Writing – original draft. HC: Supervision, Writing – original draft.
